# Enhanced De Novo Assembly of High Throughput Pyrosequencing Data Using Whole Genome Mapping

**DOI:** 10.1371/journal.pone.0061762

**Published:** 2013-04-17

**Authors:** Fatma Onmus-Leone, Jun Hang, Robert J. Clifford, Yu Yang, Matthew C. Riley, Robert A. Kuschner, Paige E. Waterman, Emil P. Lesho

**Affiliations:** 1 Multidrug-Resistant Organism Surveillance Network and Repository, Walter Reed Army Institute of Research, Silver Spring, Maryland, United States of America; 2 Viral Diseases Branch, Walter Reed Army Institute of Research, Silver Spring, Maryland, United States of America; Beijing Institute of Genomics, China

## Abstract

Despite major advances in next-generation sequencing, assembly of sequencing data, especially data from novel microorganisms or re-emerging pathogens, remains constrained by the lack of suitable reference sequences. *De novo* assembly is the best approach to achieve an accurate finished sequence, but multiple sequencing platforms or paired-end libraries are often required to achieve full genome coverage. In this study, we demonstrated a method to assemble complete bacterial genome sequences by integrating shotgun Roche 454 pyrosequencing with optical whole genome mapping (WGM). The whole genome restriction map (WGRM) was used as the reference to scaffold *de novo* assembled sequence contigs through a stepwise process. Large *de novo* contigs were placed in the correct order and orientation through alignment to the WGRM. *De novo* contigs that were not aligned to WGRM were merged into scaffolds using contig branching structure information. These extended scaffolds were then aligned to the WGRM to identify the overlaps to be eliminated and the gaps and mismatches to be resolved with unused contigs. The process was repeated until a sequence with full coverage and alignment with the whole genome map was achieved. Using this method we were able to achieved 100% WGRM coverage without a paired-end library. We assembled complete sequences for three distinct genetic components of a clinical isolate of *Providencia stuartii:* a bacterial chromosome, a novel *bla*_NDM-1_ plasmid, and a novel bacteriophage, without separately purifying them to homogeneity.

## Introduction

Next-generation sequencing (NGS) technologies, which enable the rapid generation of whole genome sequences, have revolutionized genomic research [1]–[3]. With the emergence of low-cost bench top NGS platforms, genomic studies are now being performed in translational or applied research labs instead of state-of-the art genome centers [4], [5]. Despite these technical advances, complete *de novo* sequence assembly and finishing (genome closure) continue to challenge scientists [6]. Consequently, a large number of unfinished genome draft sequences have been submitted to public databases [7]. Streamlined approaches for assembling high-quality full genome sequences are lacking, especially in the multidrug-resistant Gram-negative microbial genomics field, where *de novo* sequencing is frequently required due to the genetic diversity and dynamic genome rearrangements that occur in these organisms [8]–[10].

Among the various NGS platforms, Roche 454 GS systems are widely used for sequencing bacterial genomes [11]. The long read lengths - 300–500 bp for the FLX system and 500–800 bp for the new FLX+ system - and high sequence quality enable *de novo* assembly of reads from shotgun sequencing into large contigs. These contigs can be used for gene annotation, genotyping, and phylogeny. However, when a complete genome sequence is desired, distance information, such as that from paired-end library sequencing or extra-long read length sequencing using PacBio RS system (Pacific Biosciences, Menlo Park, CA) is required to link the *de novo* contigs [12].

WGM uses single-molecule restriction analysis to obtain information about the sizes of the restriction fragments and their physical positions along the DNA strand [13]. WGM has been used in a variety of applications, which include genotyping and phylogenetic analyses of related microbial isolates [14]–[23], detection of large genomic structural variations or rearrangements [24]–[26], and verification or quality control for assembled genome sequences [27]–[34]. A restriction-based physical genome map also has the potential to serve as a reference for the accurate ordering of NGS contigs and to facilitate closing the gaps between mapped contigs.

To date, the strategy of using WGM to scaffold sequencing contigs during *de novo* assembly has been used in projects that either employed multiple NGS platforms or used both paired-end and shotgun sequencing libraries on the Roche 454 platform [4], [26], [32], [34], [35]. Here we demonstrate that WGM can be used to direct the scaffolding of *de novo* contigs from the Roche 454 GS shotgun library sequencing to achieve the complete genome sequence of a clinical isolate of *Providencia stuartii* for which no reference genome sequence was available. This work produced the first complete *Providencia stuartii* genome sequence deposited in GenBank. WGM directed NGS assembly also facilitated the detection of extrachromosomal structures in *Providencia stuartii* MRSN 2154; namely a plasmid 178 kb in length, and a novel bacteriophage. It also yielded complete nucleotide sequences for both [36], [37].

## Materials and Methods

### Clinical bacterial isolate

MRSN 2154 is a multidrug-resistant isolate of *Providencia stuartii* recovered from the blood of a patient in Afghanistan, who ultimately succumbed to multiple infectious complications. It was subsequently found to harbor a large plasmid carrying multiple antibiotic resistance genes, including *bla*_NDM-1_, the New Delhi metallo-β-lactamase gene 1 [37], [38].

### Whole genome mapping (WGM)

WGM using the Argus system (OpGen Inc, Gaithersburg, MD) involves four steps: DNA extraction; immobilization and *in situ* restriction digestion; image capture and measurement; and map assembly and analysis. MRSN 2154 was grown on blood agar at 37°C overnight. DNA was isolated from a single colony approximately 2 mm in diameter using the Argus Sample Preparation Kit in conjunction with the Agencourt Genfind v2 DNA Isolation Kit as described by the manufacturer (OpGen Inc). This whole cell lysis protocol allows chromosomal and extrachromosomal structures to be coextracted in the same sample preparation. DNA quality and quantity was assessed using the Argus QCard Kit. In the quality control step, DNA molecules were stretched along the QCard surface, stained using Argus Stain Kit, and visualized using Argus imaging system. After verifying DNA concentration (approximately 10 molecules per frame) and length (greater than 200 kb per molecule), the sample was mapped using the Argus MapCard Kit. Briefly, single DNA molecules migrating through microfluidic channels by capillary action were immobilized on a charged glass surface. Using a restriction enzyme (NcoI) predetermined by the Argus Enzyme Chooser software module, immobilized DNA was digested *in situ* in a MapCard Processor. The Argus EnzymeChooser software identifies optimal restriction enzymes used for WGM by performing a series of virtual restriction digests of a reference genome sequence for the bacterial species or, in the case of a species without a reference genome sequence, *de novo* sequencing contigs. The software chooses an enzyme that produces an average restriction fragment size of 6–12 kb and a maximum fragment size less than 80 kb. After digestion, molecules were imaged using fluorescence microscopy on the Argus WGM system and automatically processed using its image acquisition software (OpGen Inc). This software detects cut sites in DNA molecules and calculates the length of the resulting restriction fragments by using a pixel length to kilobase conversion algorithm.

Iterative assembly from single molecules to the consensus WGRM is performed by the Argus assembly software (OpGen Inc). Downstream analysis (sequence placement) was carried out using Argus MapSolver software. DNA sequence was imported into MapSolver™ software and converted into *in-silico* maps using the same restriction enzyme as was used to generate the respective WGRM. DNA sequence contigs were aligned to the WGRM using the sequence placement function of MapSolver, which uses a dynamic programming algorithm that finds the optimal alignment of two restriction maps according to a scoring model that incorporates fragment sizing errors, false and missing cuts, and missing small fragments. The algorithm applies user-provided settings toward generating local alignments between each contig and the WGRM. DNA sequence contigs are aligned in both forward and reverse directions. MapSolver generates an alignment score for each comparison, where a higher score implies greater confidence in the alignment [39]. Alignment scores are calculated by awarding aligned fragments from the WGRM and the *in silico* map that have the exact same size in base pairs with a value of 1 and are penalized when the size or pattern deviates. Restriction fragment patterns that align will result in scores that are additive, based on the aligned fragments they contain. Therefore, longer alignments between more similar restriction patterns produced higher scores. It is recommended to use the default score of 3 for the initial replacement process. The score can be increased up to 6 to filter out spurious alignments or decreased to identify all potential alignments.

### High throughput pyrosequencing and *de novo* sequence assembly

Extraction of total DNA and high throughput pyrosequencing using the Roche GS FLX Titanium system (Roche 454 Life Sciences, Branford, CT) were previously described in detail [37]. In brief, 5 µg total DNA purified using PurElute Bacterial Genomic Kit (EdgeBio, Gaithersburg, MD) was fragmented by adaptive focused acoustic forces using the Covaris S2 system, with the shearing protocol for producing DNA fragments around 1 kb in length (Covaris, Woburn, MA). The shotgun rapid ligation library of DNA fragments was prepared, and then subjected to size selection using a 2% E-gel (Invitrogen, Carlsbad, CA) to isolate the fraction of DNA with sizes ranging from 600 to 800 bp. The library was sequenced, and the reads were subjected to *de novo* assembly using the GS Assembler software (Newbler) version 2.5.3. All contigs were used in the final sequence assembly.

### WGM-oriented assembly of scaffolds and finishing of genome assembling

**Figure 1:**
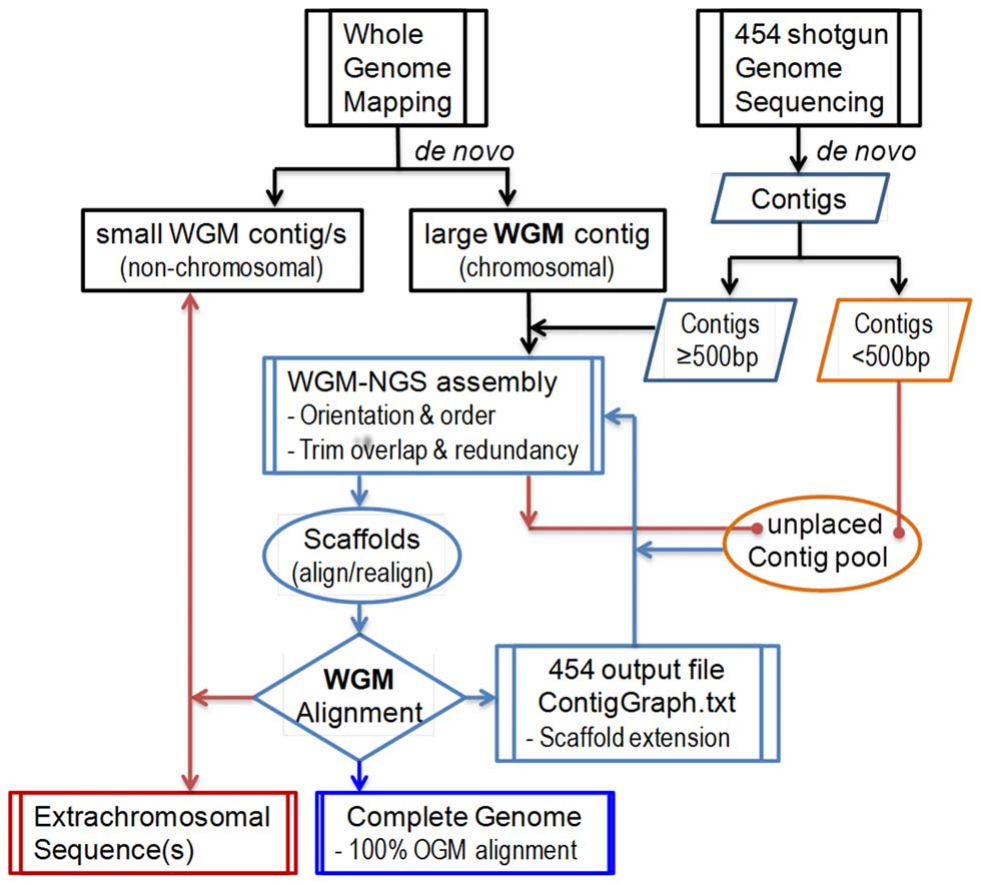
Overview of the WGM-NGS *de novo* sequence assembly process. WGM and Roche 454 pyrosequencing data are *de nov*o assembled respectively. WGM assembly software aligns single molecule restriction maps of chromosomal DNA fragments to form a contiguous contig. Depending on the size and number of the cut sites WGM can also assemble contigs for non-chromosomal elements. Roche 454 contigs that align (*in-silico* mapping) to the large WGRM contig (physical reference) are further assembled into scaffolds. Gaps are filled by extending scaffolds with sequences in the unplaced contig pool based on contig branching structure information (454 contig graph). Extended scaffolds are subsequently realigned to the WGRM. The process is repeated until high restriction pattern similarity and WGRM coverage is achieved. Contigs that do not assemble the genome are compared with the WGRM chromosomal assembly to confirm the existence of the extrachromosomal content.

**Figure 2:**
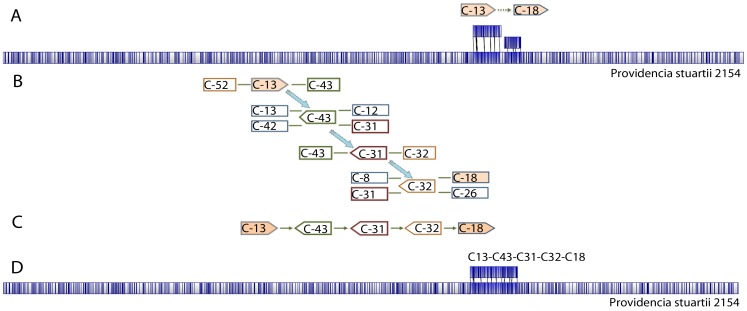
Integration of WGM alignment and 454 contig graph information for sequence assembly gap filling. The MapSolver alignment software was used with default parameters for *in-silico* alignment of sequence contigs to the WGRM. Unaligned regions are highlighted in white while alignment regions are shaded blue. Vertical lines represent NcoI cut sites. (**A**) Contigs 13 and 18 were *in-silico* digested and aligned to the WGRM to determine their orientations and the distance that separates them. There was a gap between contigs 13 and 18. (**B**) Contig branching information was used to fill the gap with contigs 43, 31, and 32. Contigs represented by boxes with right-pointing arrowheads are in the forward orientation relative to the WRGM; those represented by boxes with left-pointing arrowheads are in the reverse orientation; those represented by rectangular boxes are of unknown orientation relative to the WRGM. (**C**) Concatenation of contigs which were ordered and oriented by the WGM-enhanced gap-closure process. (**D**) Mapping the concatenated sequence to the WGRM to verify the quality of the gap filling. An *in-silico* digest of a contig is shown above the WGRM.

Whole genome restriction map assembly software aligns single molecule restriction maps of chromosomal DNA fragments, to form a contiguous contig (large contig). Molecules that are not aligned to the chromosomal map assemble as smaller contigs with smaller mapsets (usually in upper teens in number). These contigs can contain chromosomal molecules which do not match the genomic map restriction pattern, in addition to extra-chromosomal elements such as plasmids. A whole-genome NcoI restriction map was used as a template to arrange *de novo* contigs in the correct order and orientation. The workflow illustrated in Figure 1 contains the following steps: (1) The MapSolver program was used to generate *in silico* NcoI restriction maps of all NGS *de novo* contigs and align them with the MRSN 2154 WGRM. As a result, contig alignments, gaps, overlaps, and redundant contigs on the physical map were identified. (2) To fill the gaps, aligned contigs were extended using contig branching structure information from the *de novo* assembly output file 454ContigGraph.txt. The contig graph file describes the relative orientation of adjacent contigs. As an example, contig 13 has contig 43 at its 3′-end (C13→43), contig 43 has contig 12 or 31 at its 5′-end, and contig 13 or 42 at its 3′-end (12/31→C43→13/42). The contig graph information also supports the contig relationships 32→C31→43 and 18/26→C32→8/31. Therefore, the sequence between two WGM-placed contigs 13 and 18 was determined to be 13→43RC→31RC→32RC→18, where RC stands for reversed and complementary (Figure 2B–C). Additionally, contigs which did not align to the WGRM due to an inadequate number of cut sites were tested to fill the gaps. After gaps in the scaffold were filled, GS Assembler reference mapping was used to confirm continuous genome coverage across the resulting merged contigs. (3) The assembled scaffolds were aligned with the WGRM to ensure the assembly, and then joined together by eliminating any overlapped terminal sequences. (4) The assembled draft genome sequence was aligned with the WGRM to identify discordant regions. Unmapped contigs were fit into these discordant regions, and their placement was confirmed by re-alignment to the WGRM, after merging them together. Steps 1 through 4 were repeated until full genome coverage was achieved. Two additional steps (not shown in Figure 1) were taken to finish the genome assembly: Firstly, PCR amplification and Sanger sequencing were used to resolve the structure and organization of the multi-copy ribosomal RNA operons. Secondly whole-genome gene-coding annotations were used to correct reading frame shifts caused by sequencing errors in homopolymer regions. Geneious Pro v4.7 (Biomatters Ltd, Auckland, New Zealand) [40] was used for sequence editing, concatenation, translation, *in silico* restriction, and sequence alignment.

## Results

WGM technology has been used to generate high resolution whole-genome restriction map for global genomic comparison among closely related strains of microbes [41], [42]. In this study, we integrated WGM with NGS shotgun sequencing to streamline complete genome assembly of a clinically relevant bacterial pathogen.

### Whole Genome Mapping of *P. stuartii* MRSN 2154

**Figure 3:**
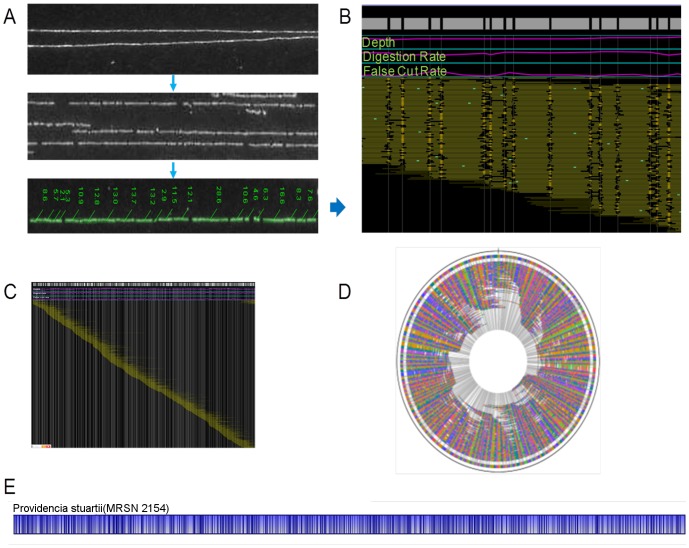
Construction of a whole-genome restriction map for *Providencia stuartii* MRSN 2154. (**A**) Genomic DNA was immobilized, *in situ* digested with NcoI enzyme, measured and converted to a digital profile. Black gaps within single DNA molecules are breaks created by restriction digestion. (**B**) DNA molecule restriction patterns were aligned to generate WGRM contigs. Each green horizontal line represents a single DNA molecule. Gray blocks represent the consensus DNA restriction fragments. Black vertical lines represent consensus restriction cut sites. (**C**) WGRM contigs aligned to create continuous coverage along the DNA strand, producing the whole-genome consensus map. (**D**) Circular representation of the linear consensus map which has an estimated size of 4.2 Mb. (**E**) The MRSN 2154 consensus genome map displayed by the MapSolver program. The circular map is illustrated in a linear view. NcoI restriction sites are indicated as vertical bars.

**Figure 4:**
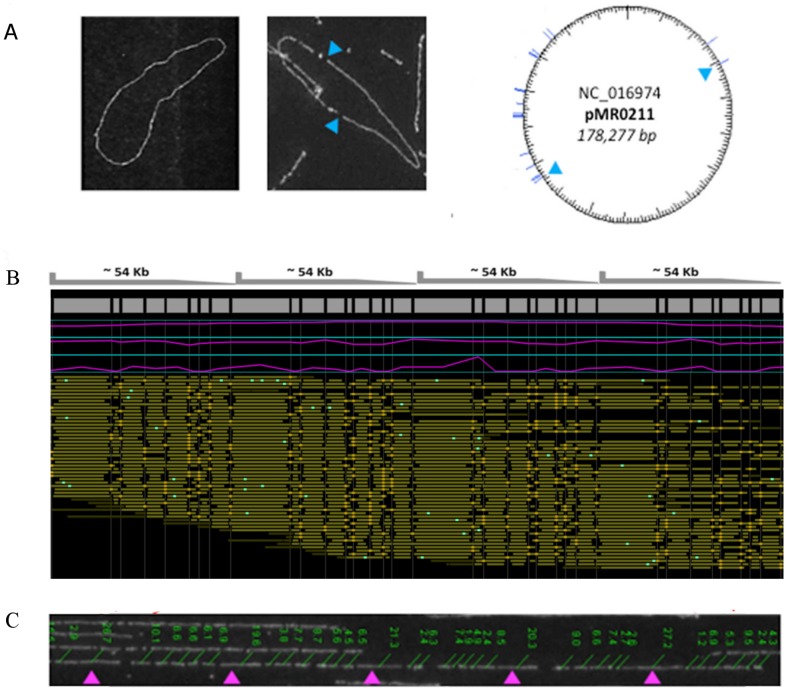
Whole genome mapping of two extra-chromosomal DNA elements in *Providencia stuartii* MRSN 2154. (**A**) A large circular plasmid with its estimated size (>160 kb) and NcoI pattern consistent with the *bla*_NDM-1_ plasmid carried by MRSN 2154. *Left*, the plasmid prior to NcoI digestion (QCard image); *center*, the NcoI-digested plasmid (MapCard image); *right*, the *in silico* NcoI restriction map for the *bla*_NDM-1_ plasmid. (**B**) A novel putative bacteriophage coexisting with MRSN 2154. The Consensus WGRM assembled for the bacteriophage suggests a repetitive structure; each repeat is approximately 54 kb in size. (**C**) Sized image of one of the DNA molecules from the bacteriophage assembly. The 20 kb NcoI fragments are indicated with the arrows in the image.

After the initial phenotypic and molecular investigation of MRSN 2154, which was the first reported *bla*_NDM-1_ containing *P. stuartii* strain [37], we applied WGM to characterize the isolate on a genomic scale. The WGM images revealed the presence of three distinct types of DNA molecules: high molecular weight chromosomal DNA fragments (Figure 3), circular plasmid molecules, and linear DNA molecules with a repetitive restriction pattern (Figure 4). A circular WGRM chromosomal assembly, with coverage ranging from 52 to 117 fold, was assembled from restriction maps of 1179 DNA molecules. The estimated genome size is 4.2 Mb (Figure 3). The plasmid was not assembled in WGM. It was estimated to be approximately 160 kb, which is similar to the sizes of some NDM-1 bearing plasmids seen in other Gram-negative bacteria [43]–[45]. The repetitive DNA structure was not assembled into the chromosome map. The coverage of this distinct repetitive element ranged from 30 to 50 fold. The size of the repeat unit was approximately 54 kb (Figure 4).

### Roche 454 sequencing and assembly of complete sequences

A shotgun rapid ligation library for MRSN 2154 was sequenced using the Roche GS FLX Titanium system. 274,932 filtered reads, totaling 82 Mb of sequence underwent *de novo* assembly using the GS Assembler software (Newbler) version 2.5.3. In total, 70 contigs ≥100 bp in size and 11 smaller contigs were assembled, with the average coverage depth being 17.5-fold (Table S1). Among the 81 *de novo* assembly contigs, 19 were assembled into the 178 kb plasmid pMR0211, which carries the *bla*_NDM-1_ gene (JN687470, NC_016974). Assembly and annotation of the pMR0211 sequence were described elsewhere [37].

**Figure 5:**
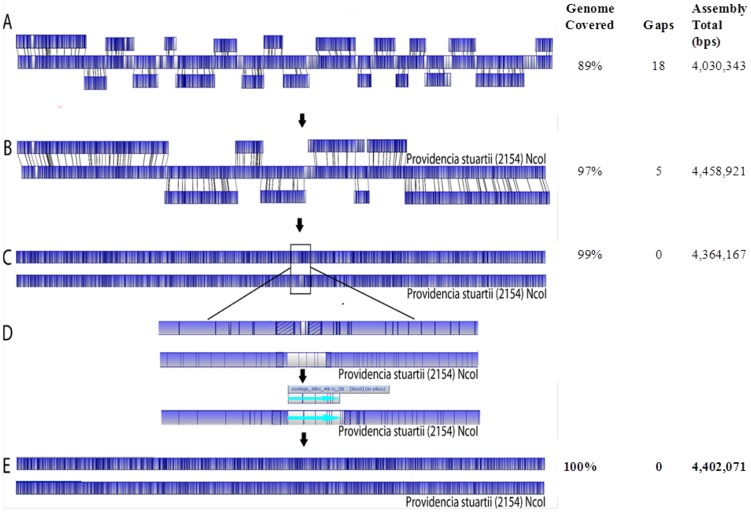
*Providencia stuartii* MRSN 2154 genome sequence assembly using the WGM-NGS approach. The MapSolver program highlights unaligned regions in white while aligned regions are shaded blue. (**A**) Contig replacement: 19 Roche 454 *de novo* contigs (>500 bp) were aligned to the MRSN 2154 WGRM in the correct orientation and order. Overlaps, redundancies, orientation and unaligned contigs were confirmed and resolved where needed. (**B**) Scaffolding by merging and extension: with the guidance of WGM and the branching structure information (454 contig graph), contigs were progressively joined to form scaffolds. Scaffolds were aligned to the WGRM to examine orientation, order, overlaps and gaps. (**C**) Alignment of first draft to the WGRM: after removal of redundant overlapping sequences, scaffolds were joined to form the first genome draft. (**D**) Correction of mismatches: An unaligned region of approximately 30 kb was identified (shown as white region) and subsequently resolved with the aid of the WGRM and branching structure information is shown in detail in Figure 2. Identified contigs were integrated in the draft. (**E**) Alignment of the complete genome sequence to the WGRM. The WGRM reference was 100% covered with highly similar NcoI restriction pattern.

The WGRM of the MRSN 2154 chromosome was used to guide assembly of contigs into the complete genome sequence (see Methods; Figure 5). Contigs 1–18 and 20, which provided 89% genome map coverage, were ordered and oriented along the WGRM (Figure 5A). The process provided information on redundant contigs, overlaps between adjacent contigs, and the approximate physical distances of gaps that required filling or closure with unplaced contigs. Redundant contigs and the overlapped terminal sequences were then removed. For the unfilled regions, the two contigs upstream and downstream of each gap were extended using contig branching structure information in the 454 *de novo* assembly contig graph file. After gap closure, the resulting 8 scaffolds were aligned to the WGRM (Figure 5B). By removing overlapping sequences, we joined these scaffolds together into a draft sequence covering 99% of the genome. Using default parameters, the draft sequence was then aligned with the WGRM for manual visual alignment. A discordance was found in one region where about 32 kb of the draft sequence was missing relative to the WGRM (Figure 5D). This disagreement between 2,437,007 bp and 2,438,048 bp was corrected using two relatively large contigs which had not been incorporated into the assembly, [contig 28 (25,715 bp) and contig 38 (4431 bp)], and the associated smaller contigs. The resulting corrected sequence showed a high level of similarity with the optical genome map. (Figure 5D).

### Determination of ribosomal RNA operons

**Figure 6:**
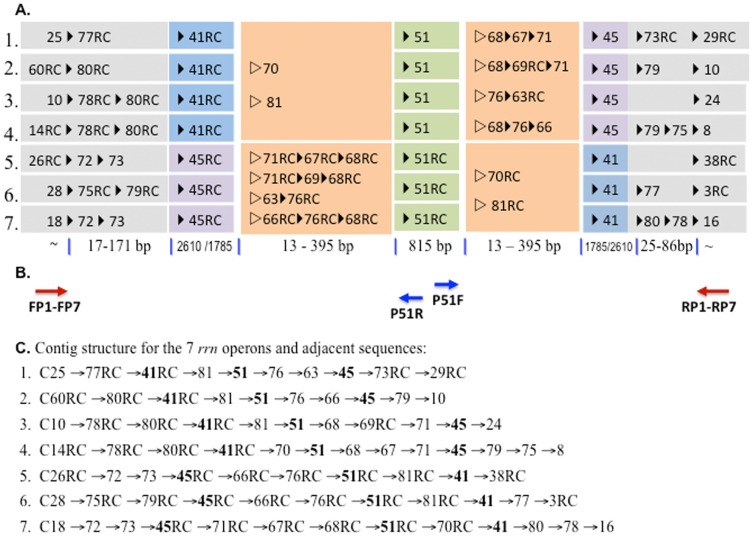
Resolved sequence structures for the 7 *rrn* operons in MRSN 2154. PCR and Sanger sequencing were used to determine the variable regions in *rrn* operons. RC, reversed and complementary. (**A**) Illustration of contig assembly for the 7 *rrn* operons and adjacent sequences. Variable regions between rRNA sequences (contigs 41, 45 and 51) were shown in *orange* boxes and indicated as orientation-undecided by the *empty arrow*. The sizes for the ribosomal and intergene regions are shown. (**B**) Schematic representation of regions to be amplified and the Sanger sequencing directions. Seven *rrn* regions were amplified by PCR using 7 pairs of primers, FP1-FP7 and RP1-RP7 which are corresponding to specific sequences flanking the *rrn* regions. Sanger sequencing outward from the conserved rRNA sequence (contig 51) in both directions with primers P51R and P51F was used to determine the intergene spacer regions. (**C**) The results for *rrn* operon sequence structures resolved by PCR amplification and Sanger sequencing.

Many bacterial genomes carry multiple *rrn* operons, which each contain one copy of the 5S, 16S, and 23S ribosomal RNA genes and a unique intergenic spacer region (ITS) [46]. We observed 7 repetitive structures in MRSN 2154 genome, which all contained the 3 rRNA genes and variable intermediate sequences (Figure 6A). However, neither WGM nor 454 sequencing can resolve the structures. The sizes of the *rrn* operons were too long to be read through by either 454 or Sanger sequencing, and the sequences were not large enough and/or the NcoI restriction patterns were not unique enough to be distinguished by WGM. We designed primers targeting upstream and downstream sequences flanking the *rrn* operons to amplify the entire *rrn* region (approximate 5.5 kb), and then we sequenced in both the forward and reverse directions from the internal rRNA gene (Table 1, Figure 6B). Among the 7 *rrn* operons in MRSN 2154, there are 4 different ITS sequence structures: an ITR in *rrn* 1; an identical ITS in *rrn* 2, 5, 6; an ITS in *rrn* 3; and an identical ITS in *rrn* 4 and 7 (Figure 6C).

**Table 1:** Oligos for amplification of *rrn* regions 1–7 and resoving *rrn* operon structures by Sanger sequencing.

*rrn* operon	Oligo	Sequence	nucleotide position	
1	C14RC-3′F	AACCCGATGATCACAAAACCGTGT	2181743	2181766
	C8-5′R	AGCGCGAACGAAAAGAAAATGCAAA	2187870	2187846
2	C26RC-3′F	AGCCGCCGAAGGGTTCAATAGG	2433678	2433699
	C38RC-5′R	TTCGAACTGGGGACCTCACCCT	2439490	2439469
3	C28-3′F	CGACAAACCGCTTCGGTGGTCA	2187795	2187774
	C3RC-5′R	AAGTGGCGGAACGGACGGGA	1192630	1192649
4	C25-3′F	AAGTGGCGGAACGGACGGGA	1192630	1192649
	C29RC-5′R	TCTGACTCCCTATAATGCGCCTCC	2433783	2433806
5	C60RC-3′F	GGCGTGATGTTCGCAACGGC	1621186	1621205
	C10-5′R	AACCGCTTCGGTGGTCAGGT	2187790	2187771
6	C18-3′F	ACATGCAAGCAGGGAAAGCAGCA	3230145	3230167
	C16-5′R	TGCAGTTCGCTGGCACAGGAT	3236226	3236206
7	C10-3′F	GGGAAAAGAAGGGGAGTGATGGAAGGT	1809355	1809381
	C24-5′R	AACCGCTTCGGTGGTCAGGT	2187790	2187771
Sanger	C51R	GGGGGACCATCCTCCAAGGCT	2184810	2184790
sequencing	C51F	CACCCTGCTCGCTCATGCCA	3232696	3232677

Each oligo name indicates a contig, contig orientation, terminus and extension direction. RC, reversed and complementary. Sequencing primers were designed to extend outwards, i.e. to sequence the regions upstream and downstream of contig 51.

Finally, the complete *P. stuartii* MRSN 2154 genome sequence was annotated using the NCBI Prokaryotic Genomes Automatic Annotation Pipeline (http://www.ncbi.nlm.nih.gov/genomes/static/Pipeline.html) and manually corrected to eliminate open reading frame shifts caused by ambiguous homopolymer calling (data not shown). After the above assembly process, a single contig, contig 22, had not been assembled into either the *P. stuartii* genome or plasmid pMR0211. To investigate whether this 58,104 bp contig is a genetic element distinct from the assembled chromosomal and plasmid genomes, the sequence was *in silico* digested with NcoI and compared with the extra-chromosomal repetitive element found by WGM. The NcoI digest of the sequence closely matched the repeat structure (Figure 4B). In addition, the contig branching information showed that this sequence did not connect to any other contigs. Furthermore, the 454Contigs.ace file, which illustrates the alignment of the reads, revealed that the sequence is continuous end-to-end. Therefore, it is either a circular DNA or a continuous sequence of tandem repeats. The sequence was then subjected to BLASTN alignment with the GenBank non-redundant nucleotide database and to BLASTX alignment with the GenBank non-redundant protein sequences database. Both results suggested the sequence represents a novel bacteriophage, which has a nucleotide sequence similarity of 65–70% with the *Enterobacter* phage Enc34 (JQ340774.1) over 61% of its length. The evidence thus suggested that there was a bacteriophage co-existing with the clinical isolate MRSN 2154. Annotation of the bacteriophage genome was performed using the xBASE server [47] and BLASTP.

### Deposition of nucleotide sequences in GenBank

The assembled complete sequences for *Providencia stuartii* MRSN 2154 genome, the *bla*_NDM-1_ plasmid, and the putative bacteriophage were deposited in GenBank under accession numbers CP003488 and NC_01773, JN687470 and NC_016974, and JX296113 respectively.

## Discussion

In this study, WGM provided both global and fine scale genomic structures that facilitated *de novo* assembly of complete bacterial genome sequences using rapid shotgun Roche 454 sequencing. We identified and sequenced three full genomic structures from a clinically important bacterial isolate. Notably, the two extrachromosomal elements, a 178-kb plasmid and a 58-kb bacteriophage, were sequenced and fully assembled without being purified to homogeneity. The quality of the assembly and the correct assignment of contigs in each genome were confirmed not only by the alignment with the WGRM profiles (Figure 5) but also by the consistency of sequence coverage depth for each contig versus the actual copy number in the final sequences (Table S1).

We demonstrated that the use of WGM simplified and streamlined NGS data assembly, by sorting and orientating *de novo* assembly contigs in the proper physical order. For laboratories that own the Argus system and can run multiple samples on a single map card [48] ,our technical approach is useful and cost-effective. The method eliminates the need for time-consuming and sophisticated paired-end sequencing, and it is functional with limited bioinformatics resources. WGM laboratory processes, image acquisition, data processing, and map assembly can be done in one to three days. In contrast, it may take several days to construct one or more paired-end libraries which can link sequences with a 3 kb, 8 kb or 20 kb span. WGM directed sequence assembly provides multiple enhancements over traditional methods: (1) it facilitates the identification of extrachromosomal DNA elements; (2) it provides an assessment of DNA molecule size and configuration (circular or linear, with a repetitive pattern for repeats); (3) it enables the detection of structural variations (large-scale rearrangements, inversions, tandem repeats) relative to reference sequences; (4) it provides clear information regarding the position and size of gaps and mismatches in the assembly; and (5) it produces a whole-genome restriction map to serve as a reference for verification and quality control of the genome assembly. WGM-NGS represents a feasible technical approach for interrogating microbial genomes from delineation of genomic components and a landscape for construction of a scaffold and finishing the complete sequence.

Next-generation sequencing platforms will become increasingly available to all types of investigators and laboratories, including clinical, non-research laboratories, as the price and the operating costs continue to decrease [5]. The procedure we described used the Roche GS FLX Titanium system and simple shotgun sequencing. With the long read length from this pyrosequencing platform, fewer than 100 *de novo* assembly contigs can be expected for a bacterial genome. With the new FLX+ system, the read length will almost double, so we expect a smaller number of contigs and a shortened time for the genome finishing process. However, to take full advantage of this technology, strategies are needed to expedite or enhance final assembly and quality in order to facilitate sensitive and precise detection of genomic variations [4].

Interestingly, the total DNA extract for the novel clinical isolate MRSN 2154 contained three distinct genetic structures at approximate equal copy number (Table S1). With the combination of WGM and NGS, we were able to sequence them altogether in a single run and assemble all three complete sequences. In our study, fine adjustments during contig scaffolding were accomplished through the combined use of OpGen MapSolver v3.2.3, Newbler assembler, and Genious and NCBI BLAST tools. A limitation of this approach is that labor-intensive and error-prone manual manipulations are still required. Further improvement will necessitate an integrated bioinformatics approach to streamline and standardize the WGM-directed sequence assembly [4], [35]. Such an approach could consist of first round placement of unique contigs using the WGRM as reference, second round placement of contigs with lower alignment scores to fill the unaligned WGRM regions, then gap closure based on contig branching structure, followed by merging overlapped sequences, and lastly, fine adjustment at joint regions by sequence remapping using original reads. Using additional enzymes in WGM to create a differential WGRM could also help resolve repetitive genomic structures and extrachromosomal elements with variable GC content and cut patterns. Currently the analysis software (MapSolver) is not publically available. Alternative tools can be developed by labs with bioinformatics expertise to manipulate the WGM data [35]. We expect that as the WGM technology becomes more accessible and accepted as the norm for sequence assembly QC, free software will become available for researchers.

In addition to the clinical pathogen described in this report, we have incorporated WGM data in the genome sequencing of other clinically relevant isolates of various species, including methicillin resistant *Staphylococcus aureus*, *Staphylococcus haemolyticus*, *Acinetobacter baumannii*, *Klebsiella*, non-equi *Rhodococcus* species, and a putative novel *Bartonella* pathogenic species. With improved integrative NGS-WGM scaffold-building software, the strategy presented here may provide high resolution microbial genotyping data useful for diagnosis and pathogen discovery in clinical settings.

## Supporting Information

Table S1 Summary of de novo contigs.
(XLSX)
